# Infectious Disease Risk Associated with Contaminated Propofol Anesthesia, 1989–2014^1^

**DOI:** 10.3201/eid2206.150376

**Published:** 2016-06

**Authors:** Andrés Zorrilla-Vaca, Jimmy J. Arevalo, Kevin Escandón-Vargas, Daniel Soltanifar, Marek A. Mirski

**Affiliations:** Universidad del Valle School of Medicine, Cali, Colombia (A. Zorrilla-Vaca, K. Escandón-Vargas);; Fundación Universitaria de Ciencias de la Salud, Bogota, Colombia (J.J. Arevalo);; Royal Free Hospital, London, United Kingdom (D. Soltanifar);; Johns Hopkins University School of Medicine, Baltimore, Maryland, USA (M.A. Mirski)

**Keywords:** Propofol, 2,6-diisopropylphenol, phenol, anesthesia, lipidemulsion, soybean, phosphatide, glycerol, edetate disodium, EDTA, fospropofol, lipophilic, intravenous, contamination, hospital infection, iatrogenic, nosocomial, safety, outbreak

## Abstract

Administration of propofol, the most frequently used intravenous anesthetic worldwide, has been associated with several iatrogenic infections despite its relative safety. Little is known regarding the global epidemiology of propofol-related outbreaks and the effectiveness of existing preventive strategies. In this overview of the evidence of propofol as a source of infection and appraisal of preventive strategies, we identified 58 studies through a literature search in PubMed, Embase, and Lilacs for propofol-related infections during 1989–2014. Twenty propofol-related outbreaks have been reported, affecting 144 patients and resulting in 10 deaths. Related factors included reuse of syringes for multiple patients and prolonged exposure to the environment when vials were left open. The addition of antimicrobial drugs to the emulsion has been instituted in some countries, but outbreaks have still occurred. There remains a lack of comprehensive information on the effectiveness of measures to prevent future outbreaks.

## Abstract 2

Transmission of illness to 144 patients, resulting in 10 deaths, has been linked to extrinsic contamination.

Globally, propofol is the most frequently used intravenous (IV) anesthetic for the induction and maintenance of general anesthesia (*1*). The chemical in propofol, 2,6-diisopropylphenol, is insoluble in aqueous solutions, so the solution is formulated as a nonpyrogenic emulsion containing soybean oil, purified egg phosphatide, and glycerol. This anesthetic has several favorable characteristics as a hypnotic agent, including rapid onset and elimination times, predictability and ease of titration, and a strong overall safety profile (*1*). Despite these benefits, propofol has been associated with the occurrence of healthcare-related infections (*2*–*4*). The potential to cause infections has been attributed to the lipophilic nature of propofol formulations, a medium that strongly supports extrinsic bacterial growth at room temperature (*5*).

In 1989, the US Food and Drug Administration (FDA) approved propofol as an induction agent for general anesthesia. Since then, numerous reports of propofol-related infections have generated strong concern among public health officials (*6*), leading to the institution of strict aseptic handling protocols and, in some countries, the additional requirement of instilling antimicrobial additives to propofol formulations. In many countries, however, no such standards have been adopted, largely because of the additional costs involved and the argument that insufficient evidence exists for the effectiveness of such antimicrobial additives. In the United States, the American Society of Anesthesiologists and the Centers for Disease Control and Prevention have jointly recommended strict adherence to aseptic handling protocols of propofol (*7*).

There continues to be a lack of awareness of the occurrence of infections related to propofol use among healthcare providers (*6*). To our knowledge, no previous review has evaluated the characteristics of propofol-related outbreaks and the evidence supporting the use of existing preventive strategies. The aim of this article is to present an overview of the evidence of propofol as a source of healthcare-related infections.

## Selection Criteria for Studies

We reviewed studies that reported on the occurrence of propofol-related infections in human subjects: single case reports, case series, retrospective chart reviews, cross-sectional studies, prospective follow-up studies, and registries published in the form of short communications or original contributions. We also reviewed laboratory studies reporting on propofol as a microbiological reservoir and studies evaluating the effectiveness of bacterial growth retardants in propofol formulations. Studies reporting on propofol-related infections in animals were excluded, as were reports found in newspaper articles and government Internet sites. The latter sources, because they are not peer-reviewed articles, provide insufficient evidence for the association between propofol and infectious events.

## Search Methods for Identification of Studies

We identified appropriate articles by searching PubMed, Embase, and Lilacs for reports published during January 1, 1989–September 30, 2014. The search was limited to articles published in or after 1989, which is the year propofol was introduced to clinical practice. The electronic search strategy for PubMed was “(propofol OR Diprivan) AND (infection OR outbreak OR contamination).” The search strategy was translated in accordance to the other database Boolean operators and was not limited by language. For this study, outbreak was defined as >2 cases.

## Study Selection

The titles and abstracts retrieved during the literature search were screened by 2 co-authors (A.Z.V., K.E.V.) independently for inclusion criteria. The full text of the selected studies was retrieved and related reference lists screened to identify additional publications. Disagreements on the selection of studies were solved by a third co-author (D.S.).

### Data Extraction and Management

We stratified selected articles into 4 categories. 1) Features of propofol-related outbreaks worldwide (e.g., year, geographic localization, type of procedure, route of propofol contamination, type of microorganism isolated, number of cases, and number of deaths) were compiled for each outbreak during 1989–2014. Outbreak reports published more than once were occasionally encountered; only the most representative study was included to prevent data duplication. In addition, we took a conservative approach in extracting data from single cases if strong associations between propofol exposure and the infectious event were reported. 2) Laboratory-based evidence of propofol as a microbiological reservoir was retrieved regarding the frequency of contaminated propofol syringes, vials, or infusion lines used in operating rooms (ORs) or intensive care units (ICUs). 3) Epidemiologic evidence concerning the risks of infections associated with propofol was confirmed by case-control, cohort, and clinical trial studies (Technical Appendix). 4) Studies on propofol formulations were used for evaluation of the data suggestive of a reduction in microbial growth associated with specific propofol formulations.

**Figure 1:**
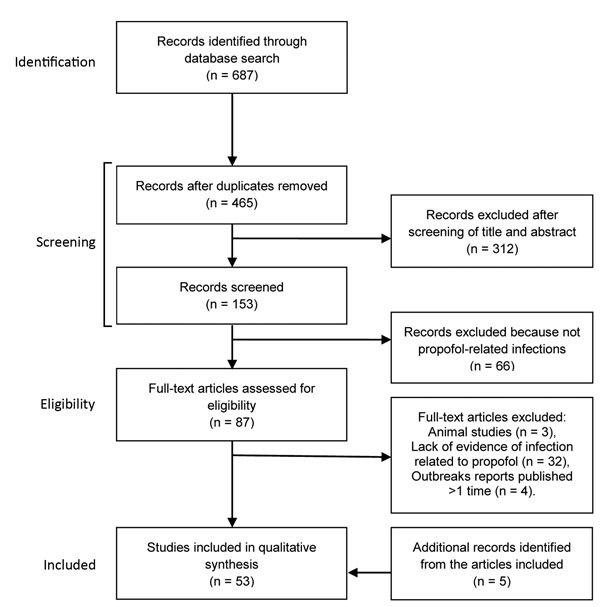
Flowchart of the selection of studies of infectious disease risk associated with contaminated propofol anesthesia, 1989–2014

We screened 465 abstracts and chose 53 to examine: 25 outbreak reports, 3 reports of single cases, 7 laboratory-based studies on propofol, 10 analytical studies that supported the healthcare effect of contaminated propofol in terms of the risk for infection, and 8 studies of the alternative propofol formulations (Figure 1). We retrieved an additional 5 articles by using references cited in 3 of the initial 53 articles to expand on specific points. 

### Worldwide Occurrence of Outbreaks Associated with Propofol-Based Anesthesia

**Figure 2:**
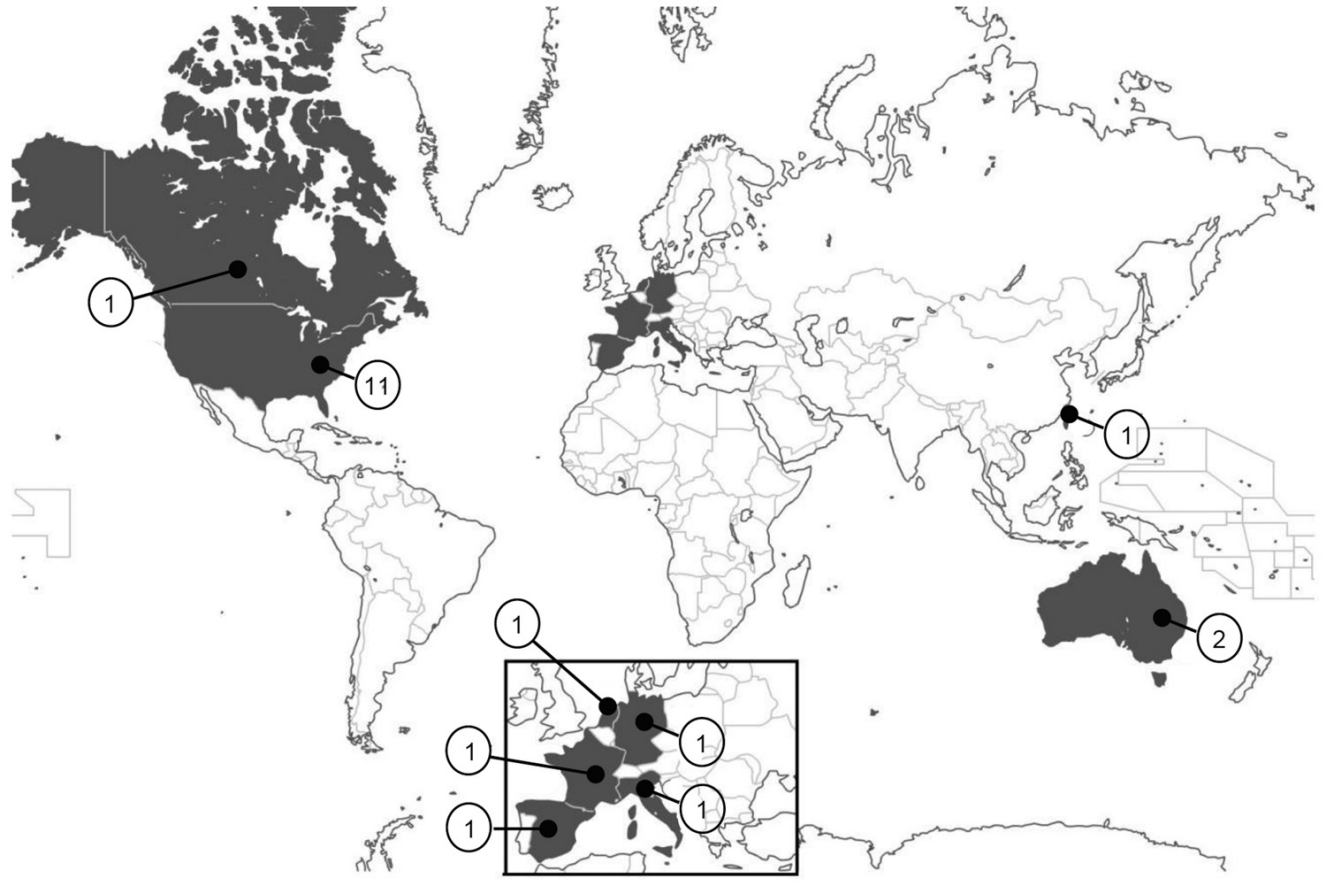
Geographic distribution of propofol-related infectious disease outbreaks worldwide, 1989–2014. Values indicate number of outbreaks for each country.

The risk for postoperative infection depends on a variety of factors, including wound class (i.e., clean, clean-contaminated, contaminated, or dirty), the condition of the patient, type and length of surgery, use of antimicrobial drugs, and perioperative events. During the past 2 decades, several episodes of sepsis worldwide have been reported to be associated with propofol administered by syringe injection or used as a continuous infusion (*2*–*4*,*6*,*8*–*15*) (Table 1). These cases were reported in industrialized countries; no outbreaks have been documented in developing or low-income countries, such as those in Latin America, Africa, or Asia (Figure 2), likely as a consequence of deficiencies of surveillance programs and poor data acquisition regarding the frequency of contaminated propofol. Propofol-associated infections likely occur in developing countries with relatively higher frequency than in industrialized countries, related to the common problem of economic restraints and reduced use of universal precautions within the healthcare systems, leading to reuse of syringes and use of vials for multiple patients.

**Table 1:** Summary data of iatragenic disease outbreaks associated with contaminated propofol reported worldwide, 1989–2014*

Location†	No. outbreaks	Duration, d‡	Year§	Type of infection	Type of surgery	Microorganism¶	No. cases	No. (%) deaths	Ref.
California, USA	1	8	1990	SSI	ND	*Staphylococcus aureus*	5	ND	(*6*)
Illinois, USA	1	5	1990	BSI, endophthalmitis	Endarterectomy, arthroscopy, dilation and curettage	*Candida albicans*	4	0	(*6*)
Maine, USA	1	2	1990	BSI	ND	*Moraxella osloensis*	2	0	(*6*)
Michigan, USA	1	14	1990	BSI, SSI	Orthopedics, gynecology, biopsy	*S. aureus*	13	ND	(*6*)
Houston, Texas, USA	1	65	1990	BSI, SSI, endophthalmitis	ND	*S. aureus*	16	2 (12.5)	(*2*)
United States	1	11	1990	BSI	General, urology, gynecology	*Enterobacter agglomerans*	4	0	(*2*)
United States	1	16	1992	BSI, SSI	Orthopedics	*Serratia marcescens*	6	0	(*2*)
United States	1	7	1992	ND	Gynecology	ND	4	0	(*2*)
Paris, France	1	0.33	1994	BSI	ND	*Klebsiella pneumoniae*	4	0	(*10*)
Atlanta, Georgia, USA	1	1	1997	BSI	Electroconvulsive therapy	*S. aureus*	5	1 (20)	(*8*)
Reggio Emilia, Italy	1	1	2001	Hepatitis C	Gynecology	HCV	5	0	(*11*)
Toronto, Ontario, Canada	1	ND	2001	BSI, SSI	Orthopedics, gastrointestinal, vascular, neurosurgery, pulmonary	*S. marcescens*	7	2 (28.6)	(*4*)
Berlin, Germany	1	ND	2002	BSI	ND	*E. cloacae*	4	2 (50)	(*3*)
Melbourne, Victoria, Australia	2	2	2003	Hepatitis C	Arthroscopy	HCV	6	ND	(*9*)
Las Vegas, Nevada, USA	1	2	2008	Hepatitis C	Endoscopy	HCV	9#	1 (11.1)	(*13*)
Alicante, Spain	1	ND	2010	Systemic candidiasis, endophthalmitis	Endoscopy	*C. albicans*	27	0	(*14*)
New York, USA	1	2	2010	Hepatitis C and B	Endoscopy	HCV, HBV	12	ND	(*12*)
Rotterdam, the Netherlands	1	2	2010	BSI, SIRS	Orthopedics, gynecology	*K. pneumoniae, S. marcescens*	7	2 (28.6)	(*15*)
Hsinchu, Taiwan	1	1	2013	Endotoxemia	Endoscopy, colonoscopy	ND	4	0	(*16*)
Total	20						144	10 (9.3)**	

*Outbreak, >2 cases; ND, not described in publication; BSI, bloodstream infection; SSI, surgical site infection; HCV, hepatitis C virus; HBV, hepatitis B virus; ref., reference; SIRS, systemic inflammatory response syndrome. †Location where the outbreak emerged. ‡Duration of the outbreak. §Year of publication. ¶Causative microorganism implicated in outbreak. #Results of HCV tests of 60,000 persons (who underwent procedures requiring anesthesia at the same clinic from March 1, 2004 through January 11, 2008) are pending. The health department identified an additional 106 infections that could have been linked to the multi-dose vials of propofol. (http://www.cdc.gov/hepatitis/Outbreaks/HealthcareHepOutbreakTable.htm). **Death rate was estimated taking into account only the published outbreaks with mortality data reported (n = 108).

Since this anesthetic was introduced, 20 propofol-related infectious disease outbreaks have been reported worldwide, affecting 144 patients and resulting in 10 deaths; these outbreaks have lasted between 8 hours and 65 days (Table 1). However, many outbreaks related to propofol are likely undocumented and such reports do not reflect ongoing sporadic infections that are likely to be linked to propofol use.

Outbreaks have been associated with widely diverse types of procedures in both intensive care units (ICUs) and operating rooms (ORs) (Table 1). Although no specific clinical procedure has been causally related to propofol-related infectious outbreaks, endoscopic procedures have been the most frequently associated with propofol-related infections during the past 20 years.

Contrary to some healthcare perceptions, none of the reported outbreaks to date have been correlated with intrinsic batch-contamination of propofol. Nevertheless, some peer-reviewed reports of manufacturing deficiencies exist, as well as >2 outbreaks probably linked to intrinsic contamination; however, the latter were published on government Internet sites and therefore are not included in this review. A US government report traced 41 cases of infection in 2009 to 1 contaminated batch (http://www.fda.gov/Safety/Recalls/ArchiveRecalls/2009/ucm172474.htm); 9 other possible cases were traced in Australia in 2014 (https://www.tga.gov.au/alert/propofol-provive-and-sandoz-propofol-1-emulsion-injection-all-sizes-and-all-batches-update-3).

**Figure 3:**
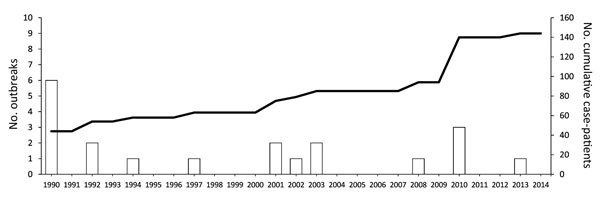
Timing of propofol-related infectious disease outbreaks worldwide during 1989–2014. An outbreak was defined as >2 cases. Dashed line indicates cumulative no. case-patients (secondary y-axis).

Distribution of propofal-related outbreaks has been widespread geographically (Figure 2) and temporally (Figure 3). The United States has reported 11 outbreaks, the highest number of outbreaks during the assessed period, averaging 1 every 2 years and accounting for 55% of all reported outbreaks worldwide. According to the list of healthcare-associated hepatitis B and C virus (HBV and HCV, respectively) outbreaks reported by the US Centers for Disease Control and Prevention during 2008–2014, the coincident recent exposure to propofol was considered a factor leading to the screening of >60,000 patients (http://www.cdc.gov/hepatitis/Outbreaks/PDFs/HealthcareInvestigationTable.pdf).

In a 2003 study of the literature on hospital-acquired infections worldwide, Vonberg and Gastmeier calculated a mortality rate of 13.8% related to administration of propofol (*17*). The data we collected indicate an estimated mortality rate in propofol–associated infections of ≈9.3% (range 0%–50%) (Table 1). This value only summarizes the current published literature describing propofol–related outbreaks, and thus it may not represent the true magnitude of the problem (*2*). According to an inspection of data held by AstraZeneca, 345 cases of postoperative infections or febrile syndrome occurred after propofol use in the United States during November 1989–November 2004; unfortunately, data for these cases were archived and not published (*2*,*18*).

### Mechanisms of Contamination of Propofol Formulations

Microbiological contamination of propofol lipid emulsions may occur from the environment either during manufacture (intrinsic) or after vial opening (extrinsic), the latter of which is the most frequent. The horizontal transmission of pathogens in anesthesia begins with the breach of handling precautions by anesthesia providers of devices or drugs. Frequently neglected precautions during the induction and maintenance of anesthesia include hand hygiene and protection against incidental propofol contact with the environment (*19*–*21*). In addition, surreptitious use of IV anesthetics by drug-addicted healthcare workers could raise the risk for extrinsic contamination (*22*). Other factors that may potentially affect the sterility of propofol in clinical use include preparation of multiple syringes for use throughout the day; re-use of vials, syringes, or infusion-pump lines on >1 patient; use of opened ampules longer than recommended by the manufacturer; and failure to wear sterile gloves during handling of propofol and to dispose of them after each contact.

The most common reservoirs associated with extrinsic contamination of propofol are syringes or micro-droppers, vials, and IV stopcock dead space. Syringes or micro-droppers have been implicated in most outbreaks (*23*–*27*). Propofol vials have been demonstrated to be a reservoir for microbes when contents are exposed to the environment (*28*). Delays in administration after propofol vials have been opened are a recognized risk factor; the degree of contamination of an opened vial may increase by 20%–26% after 12 hours (*29*). Propofol is available in vials of various volumes; a typical 20-mL vial contains 200 mg, and 50- and 100-mg vials are available. It is believed that administering a dose >200 mg to an adult in an OR will increase the probability of using an additional vial as a multi-dose vial for >1 patients. IV stopcock dead space has been shown to provide a potential route of entry for pathogenic, multidrug-resistant bacteria in infusion lines (*20*,*27*).

A number of microorganisms are associated with propofol in clinical- and laboratory-based studies at varying frequencies (Table 2). We report the specific pathogens that have been associated in several outbreaks (*2*–*4*,*6*,*8*–*10*,*12*–*15*,*30*,*31*), as well as all of the transmissible microorganisms that have been observed in contaminated propofol in syringes, vials, and infusion lines (*23*,*25*,*27*,*30*,*32*). We also describe 3 reported cases of septic shock related to propofol (*33*,*34*).

**Table 2:** Microorganisms identified in propofol anesthesia-related iatragenic infection outbreaks, single cases, or laboratory-based studies of syringes, vials, or infusion lines*

Category and microorganism	Type of infection	% Infections†	References
Gram-positive bacteria		27.08	
* Staphylococcus aureus*	BSI, SSI	27.08	(*2*,*6*,*8*,*30*)
* S. epidermidis*‡	–	–	(*23*,*27*,*30*,*31*)
MRSE§	SSI	–	(*31*)
* Streptococcus salivarius*‡	–	–	(*22*)
* Enterococcus faecalis‡*			(*32)*
* Micrococcus* sp.‡	–	–	(*23*,*25*,*27*)
* Corynebacterium* sp.‡	–	–	(*23*)
* Bacillus* sp.‡	–	–	(*23*,*25*)
* Diphtheroids* sp.‡	–	–	(*25*)
* Kocuria* sp.‡	–	–	(*27*)
Gram-negative bacteria		20.14	
* Serratia marcescens*	BSI, SSI	9.72	(*2*,*4*,*15*,*30*)
* Enterobacter cloacae*	BSI	2.78	(*31*)
* E. agglomerans*	BSI	2.78	(*2*)
* Pseudomonas cepacia*§	BSI	–	(*34*)
* P. aeruginosa*§‡	SSI	#	(*30*,*33*)
* Escherichia coli*§	BSI	–	(*35*)
* Klebsiella pneumoniae*	BSI	3.47	(*10*,*15*)
* Moraxella osloensis*	BSI, SSI	1.39	(*6*)
* Acinetobacter* sp.‡	–	–	(*27*)
Fungus		21.53	
* Candida albicans*	BSI, SSI	21.53	(*2*,*6*,*14*,*30*)
Viruses		22.53	
HCV	Hepatitis C	18.06#	(*9*,*11*–*13*)
HBV	Hepatitis B	4.17	(*12*)

*Outbreak, >2 cases; BSI, bloodstream infection; SSI, surgical site infection; MRSE, methicillin-resistant *Staphylococcus epidermidis*; HCV, hepatitis C virus; HBV, hepatitis B virus; dashes indicate no infections identified †Percentage of infection estimated among the total of victims involved only in outbreaks in which a pathogen was identified (n = 131). In total, 9.03% of the patients reported in the outbreaks had no microorganisms identified, in part because the cultures were obtained after administration of antimicrobial drugs. ‡Microorganisms that have been identified by culture of residual propofol after clinical use but so far have not been involved in propofol-related outbreaks or infection associated with propofol. §MRSE, *P. cepacia, P. aeruginosa*, and *E. coli* have been identified in case reports of infection and septic shock, but so far have not been involved in propofol-related outbreaks. #*P. aeruginosa* and HCV have been implicated in outbreaks in Catalonia and Galicia, Spain. However, these reports appeared in newspapers and because of that were not included in this synopsis article (http://elpais.com/diario/2011/03/05/sociedad/1299279606_850215.html and http://elpais.com/diario/2011/03/09/sociedad/1299625207_850215.html).

Propofol is an excellent medium not only for bacterial growth but also for fungal infections, which have been associated with propofol use particularly when poor hygienic standards are observed during the administration. Viral infections with HCV and HBV have also been demonstrated, possibly explained by the viral stability offered by propofol emulsions (*9*,*11*,*13*). Overall, ≈23% of the published infection outbreaks associated with propofol were caused by HCV (18.1%) and HBV (4.2%); 21.5% by *Candida albicans*; and 47.2% by bacteria (gram-positive 27.1%, gram-negative 20.1%). In the remaining reports, no microorganisms were identified, possibly as a consequence of concurrent antimicrobial drug therapy. The most frequent pathogens associated with propofol-related outbreaks, in order of frequency, were *Staphylococcus aureus* (39/144), *Candida albicans* (31/144), and HCV (26/144).

Laboratory-based (Table 3) and epidemiologic (online Technical Appendix Table 1) microbiological studies have demonstrated that the production of bacterial endotoxins is greatly enhanced by propofol solutions. Case reports of endotoxemia associated with the use of contaminated propofol have also been published (*5*,*34**,**35*). 

### Frequency of Contaminated Propofol Used in ICUs and ORs

Microbiological observations of opened propofol vials were reported in the first studies that identified bacterial growth in propofol, and observational studies on propofol formulations have determined the proportion of extrinsic contamination (Table 3). In 1994, Farrington et al. established an incidence rate of 6% (3/50) of contaminated propofol syringes in an ICU (*31*). Webb et al. retrospectively observed similar results in a different ICU setting with an incidence of 5.9% (18/302) (*23*). Soong observed a lower propofol contamination incidence (3.0%) in ORs and also noted an association between postoperative infections and vials from which multiple patients were medicated (*24*); Bach et al*.* found similar results (*30*). In 1995, McHugh and Roper reported an incidence of 6.3% (16/254) of infection when propofol was administered from vials but did not find that delays in the administration of propofol were associated in any increased likelihood of bacterial contamination (*25*). Cole et al. recorded the incidence of contamination as high as 17.3% (26/150) in propofol found in stopcock dead space (*27*). 

**Table 3:** Summary of studies of syringes, vials, infusion lines, and IV stopcock dead spaces for contamination after clinical use to administer propofol anesthesia*

Object† and study, year (reference)	Country	Antimicrobial agents‡	Hospital unit§	Crude % contaminated propofol (no. contaminated/no. tested)
Syringes				
Farrington et al., 1994 *(31*)	United Kingdom	No	ICU	6.0 (3/50)
Bach et al., 1997 (30)	Germany	No	OR	4.8 (8/168),¶ 5.1 (19/376)#
Webb et al., 1998 (23)	Australia	ND	ICU	5.9 (18/302)
Total				5.4 (48/896)**
Vials				
McHugh et al., 1995 (25)	New Zealand	No	OR	6.3 (16/254)
Soong et al., 1999 (*24*)	Australia	ND	OR	3.0 (3/100)
Zorrilla-Vaca et al., 2014 (*32*)	Colombia	No	OR	6.1 (12/198)
Total				5.6 (31/552)**
Infusion systems				
Bach et al., 1997 (*30*)	Germany	No	ICU	4.5 (10/224),¶ 1.6 (5/318)#
Lorenz et al., 2002 (*26*)	Austria	No	OR	11.3 (9/80),†† 8.8 (7/80)‡‡
Total				4.4 (31/702)**
IV stopcock dead spaces				
Cole et al., 2013 (*27*)	United States	Yes	OR	17.3 (26/150)

*ICU, intensive care unit; OR, operating room; ND, not described in publication; IV, intravenous. †Clinical object from which residual propofol was taken to be cultured after clinical use. ‡Use of propofol formulations with antimicrobial additives. §Hospital unit where the studies were conducted. ¶Results of a first study period during February 1­–October 31, 1992. #Results of a second study-period from December 1, 1994, through March 31, 1995. **Total crude percentage of contaminated propofol for each kind of object (syringes, vials, infusion systems). ††Proportion of propofol contaminated, following the manufacturer’s handling recommendations. ‡‡Proportion of propofol contaminated, following a modified propofol handling protocol. (i.e., refilling empty syringes and renewing only the infusion line to the patient).

The distribution of instructions for aseptic measures for handling propofol has shown to reduce the rate of contaminated propofol. Lorenz et al. reported that after a specific protocol for handling propofol was introduced and adhered to, a reduction in extrinsic contamination was achieved when compared with only adhering to the manufacturer’s recommendations (8.8% versus 11.3%) (*26*). That protocol included additional aseptic precautions, such as refilling empty syringes for use on multiple patients by using a 3-way stopcock and replacing only the infusion line to the patient. Data from a study performed in a high-complexity hospital in Cali, Colombia, showed substantial microbial growth of 6.1% (12/198) in residual propofol vials used in ORs at a tertiary hospital in Cali (*32*).

By collating the incidences of contaminated propofol in containers or devices, the data suggest that it is more common to encounter contaminated previously used vials of propofol (5.6%, 31/552) than in used syringes (5.4%, 48/896) and infusion systems (4.4%, 31/702). Similarly, analyzed by hospital location, the percentage of contaminated propofol is greater in ORs (7.3%, 103/1,405) than in ICUs (4.0%, 36/894), correlating well with the expected prevalence of opened vials and syringes used for bolus injections of propofol for multiple patients in the OR compared with those in the ICUs.

### Risk for Infection Data Derived from Analytical Studies

**Figure 4:**
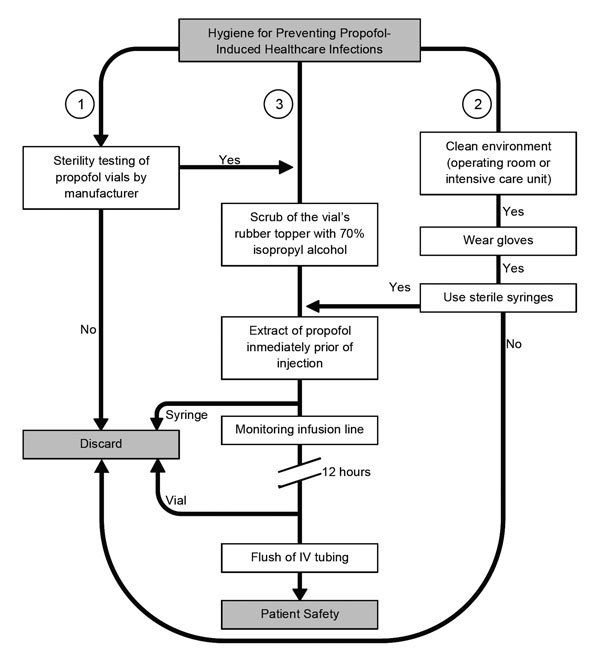
Algorithm for helping reduce the likelihood of infectious disease events when using propofol. To avoid intrinsic contamination, sufficient quality control during the manufacturers’ process is required (*1*). Personnel must be aware of the importance of performing healthcare procedures in a clean environment and the use of gloves and sterile syringes for anesthetic procedures. Syringes and needles must never be reused (*2*). Also, the aseptic technique for administration of propofol includes cleaning of the rubber bung, if present, with isopropyl alcohol, leaving it to dry. Propofol should be drawn up immediately before its use and not left standing. Intravenous (IV) infusion lines and stopcock dead spaces should be completely flushed to ensure no residual propofol remains. Vials must be discarded after opening for single use, no matter the amount of the remainder (*3*).

After we reviewed the initial microbiological studies, our interest increased in ascertaining potential links between the use of propofol infusions and the incidence of infections and sepsis after surgery. We analyzed 10 epidemiologic studies (Table 4; Technical Appendix Table 1); of these, 4 assessed the associations with infection when practitioners followed manufacturers’ instructions for propofol handling, 5 assessed the association with infection when practitioners did not follow instructions, and 1 did not report on this issue. Studies following manufacturers’ precautions stated such within the articles, but the degree of compliance of such precautions was not documented (Technical Appendix Table 2). In 4 of 5 studies (80%) during which practitioners did not follow handling precautions, high infection risk was noted. In 4 studies in which precautions were followed, 2 (50%) scored above the significant risk threshold (Technical Appendix Table 1). These findings underscore the controversy surrounding the utility of existing handling protocols and demonstrates the continued high potential of propofol as a causal factor of iatrogenic infection. Other authors have explain the susceptibility to infection because of the attenuation of the immunological activity caused by propofol infusions. More studies of specific handling protocols are required before a significant risk reduction is clearly observed. We developed an algorithmic approach that shows certain crucial measures to prevent future propofol-related outbreaks of infections (Figure 4); this approach was based on our analyses and summaries of the epidemiologic and clinical data selected.

**Table 4:** Summary of epidemiologic studies analyzing the association between infectious conditions and contaminated propofol anesthesia*

Followed manufacturers’ precautions, study, year	Type of study	Preservative-free propofol†	Other agents compared with propofol	Type of infection	Hospital unit‡	Association§
Yes						
Seeberger et al., 1998	Retrospective cohort	Yes	Thiopentone	Sepsis	OR	No
Shimizu et al., 2010	Cohort	ND	Sevoflurane	SSI	OR	Yes
Haddad et al., 2011	Nested cohort	Yes	ND	Multiple¶	ICU	Yes
Moehring et al., 2014	Case–control	ND	Fentanyl	BSI	ICU	No
No						
Bennett et al., 1995. (*2*)	Case–control and cohort	Yes	Sufentanil, alfentanil	BSI, SSI	OR	Yes
Henry et al., 2001 (*4*)	Case–control	Yes	ND	BSI, SSI	OR	Yes
McNeil et al., 1999	Cohort	Yes	Sufentanil, fentanyl, midazolam, vecuronium	Fungemia, endophthalmitis	OR	Yes
Sebert et al., 2002.	Case–control	ND	ND	BSI	OR	No
Muller et al., 2010 (*15*)	Retrospective cohort	ND	Fentanyl, midazolam	BSI, SIRS	OR	Yes
ND						
Kontopoulou et al., 2012	Case–control	ND	ND	BSI	ICU	Yes
*Complete data and full references are available in the Technical Appendix; OR, operating room; ICU, intensive care unit; BSI, bloodstream infection; SSI, Surgical site infection; ND, not described in publication; SIRS, systemic inflammatory response syndrome. †Use of propofol without antimicrobial additives. ‡Hospital unit where the studies were conducted. §Conclusion of the analytical study regarding the association between propofol exposure and infectious events. ¶Multiple infection types, including ventilator-associated pneumonia, urosepsis, BSI, catheter-related infections, and others.						

### Proposed Propofol Formulations without Risk for Infection

There are currently no propofol formulations without infection risk; however, several investigators have advocated the use of antimicrobial additives. As required by the FDA, the efficacy of such additives must retard microorganism growth to <10-fold at 24 hours after extrinsic contamination of propofol (*1*); many suggested antimicrobial agents have been rejected because of their poor efficacy, additional side effects, or higher costs (*1*). The fact that propofol is a lipid emulsion poses problems when additives are considered because admixture with other substances, especially charged species with differing partition coefficients, can interact with either the lipid phase or the emulsifying agents, resulting in emulsion destabilization. Older and newer advances on the use of additives are described in Table 5, which includes the scientific progress related to patients, clinical trials, original articles, and brief reports.

**Table 5:** Description of advantages and disadvantages of each formulation of propofol related to contamination and iatragenic infection*

Propofol formulation	Settings	Advantages	Disadvantages	FDA approval
Propofol with EDTA	Antimicrobial activity	This mixture with propofol at 0.005% wt/vol concentration has demonstrated microbial growth to be retarded to >1 log CFUs (*36*), of nearly 20 microorganisms, including 7 Gram-positive bacteria, 10 Gram-negative bacteria, and 3 yeasts (*18*). Further, incidence of propofol-related infection declined from 39 to 9 infections per year in the USA, after the introduction of EDTA into clinical use in 1996 (*18*).	Decreases serum ionized calcium levels, although statistically significant, has apparently no clinical effect (time to complete recovery, p = 0.77 [*37*]). Also, EDTA is nephrotoxic at high doses (2–3 g/d). Concern that use of an antimicrobial may cause health personnel to relax on aseptic handling practices (*1*).	Yes
Fospropofol disodium	Nonlipophilic preparation	Because of water solubility, eliminates some of the known lipid emulsion-associated disadvantages of propofol, including the risk for infection (*38*).	Minor side effects (e.g., paresthesia, hypotension). The prolonged onset of action of fospropofol (≈4–13 min, because of it must first undergo metabolism to propofol) compared with the prodrug propofol (≈40 s). Allergies caused by the accumulation of a phosphate-ester component (*38*).	Yes†
Propofol and lidocaine	Bacteriostatic activity	Experimentally causes loss of viability of several strains (*29*).	Has no sufficient retarding effect. Possibilities of micelle formation exist.	No
Benzyl alcohol	Antimicrobial activity	At low concentrations of >2%, has been used as a preservative agent.	Toxicity and presumed instability.	No
Sodium metabisulfite	Antimicrobial activity	Reduces the pain of propofol injection and has preservative properties.	Has a labeled pH of 4.5–6.4, which is different from the required pH for propofol (6–8.5) (*1*).	No
EmulSiv filter	Filter	Use of the 0.45 µm-rated filter is purported to provide protection from accidental microbial contamination, particulate contamination and entrained air while maintaining the integrity of the emulsion (*40*).	High costs, not currently available.	No
Nonlipid propofol nanoemulsion	Nonlipophilic preparation	Replaces soybean lecithin with polyethylene glycol 660 hydroxystearate as propofol carrier (*40*).	High costs, not currently available.	No

*FDA, US Food and Drug Administration. †Approved for use by the FDA only for monitored anesthesia care; however, a decision from the US Drug Enforcement Agency could be scheduled (*38*).

### Propofol with EDTA

As an antimicrobial ion chelator, EDTA exerts its effect by removing divalent and trivalent metal cations, causing rupture of the microbial cell membrane by loss of control of osmotic pressure gradients. This combination is approved by the FDA; clinical trials demonstrated antimicrobial efficacy and safety in humans (*18*,*36*). For manufacturers, EDTA is the most broadly incorporated agent in combination with propofol formulations. Despite its widespread use, some controversy remains over its selection as the optimal additive (*37*). Moreover, in several developing countries, cost remains a considerable limiting factor for the use of EDTA-containing propofol formulations (A. Zorrilla-Vaca et al., unpub. data).

### Fospropofol Disodium

A newly introduced agent, fospropofol disodium, is a water-soluble pro-drug of propofol that currently has a small evidence base for its use of 3 published clinical trials in the literature; in these studies, fospropofol was assessed for use in sedation for colonoscopies, bronchoscopies, and coronary artery bypass graft surgery, and showed an acceptable safety profile (*38*). The advantage of this drug in reducing the risk for infectious events is that it does not have a lipophilic formulation that would support bacterial growth. Nonetheless, the drug has some disadvantages that could discourage its use, such as transient paresthesias and pruritis in the perineal and perianal regions (*38*).

### Anesthetic Mixtures

Pain on injection is a common side effect of propofol. Anesthesiologists use a variety of strategies to reduce this, such as the addition of 1–2 mL of lidocaine to the propofol before injection. Lidocaine, like other amide local anesthetics, has bacteriostatic properties, which could theoretically reduce the chances of infection (*29*). It is, however, not currently known whether lidocaine has sufficient antimicrobial effect to make a clinical difference in infection rates.

### Other Additives

Benzyl alcohol at concentrations of <2% has been used as a preservative agent in propofol formulations (*39*). Despite its bacteriostatic activity, benzyl alcohol used in propofol formulations is limited by its toxicity and instability in the combination. Other additives, such as phenylmercuric nitrate, phenylmercuric acetate, chlorobutanol, and phenol have been studied experimentally with propofol; however, all of these agents were rejected because of their potential toxicity. The sodium metabisulfite–containing formulation, created originally with the aim of reducing the pain of propofol injection, has been shown to possess preservative properties. Unfortunately, it has a labeled pH from 4.5 to 6.4, which is different from the FDA requirement of a pH of 6–8.5 for propofol (*1*). The nonlipid nanoemulsion and EmulSiv filter are the most recent alternative propofol formulations (*40*). In recent studies, these formulations of propofol have attained a level of antimicrobial activity above that observed with propofol with EDTA (*40*). Costs are currently a limiting factor for their use, but these 2 options seem to provide some promise for the future if production costs decline.

## Discussion

Contaminated propofol has been implicated in several episodes of iatrogenic infection in both the outpatient and inpatient settings, as well as in both surgical and nonsurgical patients (*2*,*6*,*11*,*12*,*15*). The risk for infection arises principally because the lipophilic nature of propofol supports microbial growth when the formulation becomes contaminated (*2*,*6*). In addition, the method of intravenous administration and the preservative-free preparations still used in many countries have been implicated in promoting infection with propofol use.

More than 2 decades have elapsed since the first outbreaks of contaminated propofol-related infection emerged in the United States (*6*), and incidents of contamination-related infections persist, despite the introduction of antimicrobial formulations. Contamination and infections associated with propofol have been most commonly reported in industrialized countries, but it is likely that this phenomenon is secondary to a lack of surveillance of propofol contamination in developing countries. Management of this risk for contamination and infection can be approached by continued medical education regarding patient safety. A lack of adherence to the manufacturers’ guidelines appears to have been a causative factor in most of the episodes reported worldwide. The adherence to strict aseptic handling protocols is mandatory and more education efforts (e.g., the One and Only Campaign, http://www.oneandonlycampaign.org/) are needed to generate awareness in the healthcare community of the importance of proper propofol practices.

In summary, healthcare-associated infections linked with contaminated propofol constitute a complex public health issue that requires a multifaceted approach. Further efforts in surveillance and research are required to reduce the potential harm from contaminated propofol. Healthcare practitioners must focus on standard hygienic measures and the increased use of approved antimicrobial propofol formulations. Following these simple tenets, the risk for in-use contamination would be lowered and the safety use profile for propofol would greatly improve.

Technical Appendix Summary of analytical studies of the risk of healthcare-related infections associated with propofol and adherence by health professionals to manufacturer’s recommendations and aseptic technique.
