# Supplementary material for: Infectious Disease Risk Associated with Contaminated Propofol Anesthesia, 1989–2014
Source: Emerg Infect Dis. 2016 Jun;22(6):981–92. doi: 10.3201/eid2206.150376 (PMC4880094; doi:10.3201/eid2206.150376)
Supplement: Technical Appendix — Summary of analytical studies of the risk of healthcare-related infections associated with propofol and adherence by health professionals to manufacturer’s recommendations and aseptic technique. [file 15-0376-Techapp-s1.pdf]

Article DOI: http://dx.doi.org/10.3201/eid2206.150376

Infectious Disease Risks Associated with Contaminated Propofol Anesthesia, 1989–2014

Technical Appendix

Epidemiologic Studies of Propofol-Related Infections

With the aim of comparing the risk of infections associated with propofol when following or not following manufacturers' recommendations, we reviewed the analytical studies that have assessed the epidemiological relation between propofol exposure and healthcare-related infection. Technical Appendix Table 1 is a summary of all analytical studies found.

Data search provided 4 analytical studies following manufacturers' recommendations. In 1998, Seeberger et al conducted a retrospective cohort and failed to document significant risk of infection following the manufacturers' precautions (*1*). Recently, Moehring et al. obtained similar results (*2*). Contrary to these studies, Shimizu et al found a significant standardized infection risk following manufacturers' precautions (standardized infection ratio 4.78, 95% CI 4.30–5.27, p = 0.02) (*3*). Also, Haddad et al found statistically significant risk of intensive care unit-acquired infections after propofol infusions following aseptic technique (adjusted odds ratio [aOR] 1.89, 95% CI 1.17–3.06, p = 0.009) and furthermore, they found significant risk of ICU-acquired sepsis as well (aOR 1.91, 95% CI 1.12–3.28, p = 0.02) (*4*). In regard with this evidence, we consider that there exists a lack of sufficiently effective measures for avoiding infections secondary to propofol use.

On the other hand, 5 analytical studies, in which personnel did not follow manufacturers' guidelines, have been conducted. In 1995, Bennett et al. conducted a study in which 3 of 7 outbreaks were related to misuse of propofol (*5*). Henry et al conducted a case-control study with the aim of determining the risk factors associated with infection and

concluded that the infusion of propofol, without aseptic technique, was a significant risk factor (OR 22, 95% CI 2.1-550, $p<0.05$) (*6*). Also, McNeil et al obtained a significant infection risk (relative risk 8.8, $p = 0.048$) (*7*). Muller *et al* showed the strongest association, reporting infection in 7 out of 17 patients exposed to propofol and in none of the 18 unexposed patients ($p = 0.003$) (*8*). In particular, Sebert *et al* did not find significant risk of infection (*9*).

Another study by Kontopoulou *et al* found significant risk of infection, but the authors did not report if specific handling precautions were followed or not (*10*).

References

1. Seeberger MD, Staender S, Oertli D, Kindler CH, Marti W. Efficacy of specific aseptic precautions for preventing propofol-related infections: analysis by a quality-assurance programme using the explicit outcome method. J Hosp Infect. 1998;39:67–70. PubMed http://dx.doi.org/10.1016/S0195-6701(98)90245-6

2. Moehring RW, Lewis SS, Isaacs PJ, Schell WA, Thomann WR, Althaus MM, et al. Outbreak of bacteremia due to *Burkholderia contaminans* linked to intravenous fentanyl from an institutional compounding pharmacy. JAMA Intern Med. 2014;174:606–12. PubMed

3. Shimizu K, Hirose M, Mikami S, Takamura K, Goi T, Yamaguchi A, et al. Effect of anaesthesia maintained with sevoflurane and propofol on surgical site infection after elective open gastrointestinal surgery. J Hosp Infect. 2010;74:129–36. PubMed http://dx.doi.org/10.1016/j.jhin.2009.10.011

4. Haddad S, Tamim H, Memish ZA, Arabi Y. Association of preservative-free propofol use and outcome in critically ill patient. Am J Infect Control. 2011;39:141–7. PubMed http://dx.doi.org/10.1016/j.ajic.2010.05.027

5. Bennett SN, McNeil MM, Bland LA, Arduino MJ, Villarino ME, Perrotta DM, et al. Postoperative infections traced to contamination of an intravenous anesthetic, propofol. N Engl J Med. 1995;333:147–54. PubMed http://dx.doi.org/10.1056/NEJM199507203330303

6. Henry B, Plante-Jenkins C, Ostrowska K. An outbreak of *Serratia marcescens* associated with the anesthetic agent propofol. Am J Infect Control. 2001;29:312–5. PubMed http://dx.doi.org/10.1067/mic.2001.117043

7. McNeil MM, Lasker BA, Lott TJ, Jarvis WR. Postsurgical *Candida albican*s infections associated with an extrinsically contaminated. J Clin Microbiol. 1999;37:1398–403. PubMed

8. Muller AE, Huisman I, Roos PJ, Rietveld AP, Klein J, Harbers JB. Outbreak of severe sepsis due to contaminated propofol: lessons to learn. J Hosp Infect. 2010;76:225–30. PubMed http://dx.doi.org/10.1016/j.jhin.2010.06.003

9. Sebert ME, Manning ML, McGowan KL, Alpern ER, Bell LM. An outbreak of *Serratia marcescens* bacteremia after general anesthesia. Infect Control Hosp Epidemiol. 2002;23:733–9. PubMed http://dx.doi.org/10.1086/502003

10. Kontopoulou K, Tsepanis K, Vasiliagkou E, Katsanoulas K, Stoikou I, Antoniadou E, et al. Risk factors for *Burkholderia cepacia* complex bacteraemia among intensive care unit patients in a Greek general hospital [abstract]. Clin Microbiol Infect. 2012;18(S3):291. http://dx.doi.or/10.1111/j.1469-0691.2012.03802.x/abstract

11. King CA, Ogg M. Safe injection practices for administration of propofol. AORN J. 2012;95:365–72. PubMed http://dx.doi.org/10.1016/j.aorn.2011.06.009

Technical Appendix Table 1. Summary of epidemiologic studies analyzing the association between infectious events and propofol*

Followed manufacturers' precautions	Study, year, (reference)	Type of study	Preservative-free propofol†	Others agents compared to propofol	Type of infection in the cases	Antimicrobial drug prophylaxis‡	Hospital unit§	Conclusions
Yes								
	Seeberger et al., 1998 (*1*)	Retrospective cohort	Yes	Thiopentone	Sepsis	Yes	Operating room	Infection rate in the propofol group: 0.2% (3/1,407). Infection rate in the thiopental group: 0.2% (10/5,026), p = NS.
	Shimizu et al., 2010 (*3*)	Cohort	ND	Sevoflurane	SSI	Yes	Operating room	After matching, SSI rate in propofol group: 16.7% (14/84) and in sevoflurane group: 7.1% (6/84). SIR: 4.78 (95% CI 4.30–5.27), p = 0.02.
	Haddad et al., 2011 (*4*)	Nested cohort	Yes	ND	Multiple¶	ND	ICU	Risk for ICU-acquired infections, aOR: 1.89 (95% CI 1.17–3.06), p = 0.009. Risk for ICU-acquired sepsis, aOR: 1.91 (95% CI 1.12–3.28), p = 0.02.
	Moehring et al., 2014 (*2*)	Case-control	ND	Fentanyl	BSI	Yes	ICU	OR = 4.36 (95% CI 0.72–472.48), p = 0.19.
No								
	Bennett et al., 1995 (*5*)	Case-control and cohort	Yes	Sufentanil, alfentanil	BSI, SSI	ND	Operating room	Hospital No. 2, RR: 8.8<br>Hospital No. 3, RR: 4.5<br>Hospital No. 6, RR: 20<br>p = ND
	Henry et al., 2001. (6)	Case-control	Yes	ND	BSI, SSI	ND	Operating room	OR = 22 (95% CI 2.1–550), p<0.05.
	McNeil *et al*, 1999. (7)	Cohort	Yes	Sufentanil, fentanyl, midazolam, vecuronium	Fungemia, endophthalmitis	Yes	Operating room	RR: 8.8, p = 0.048.
	Sebert et al., 2002. (9)	Case-control	ND	ND	BSI	Yes	Operating room	OR = 1.9 (95% CI 0.47–6.3), p = 0.24.
	Muller *et al*, 2010. (8)	Retrospective cohort	ND	Fentanyl, midazolam	BSI, SIRS	Yes	Operating room	Exposed to propofol (7/17) versus unexposed (0/18), p = 0.003.
ND								OR = 5.23 (95% CI 2.2–8.46), p = 0.012.
	Kontopoulou et al., 2012. (10)	Case-control	ND	ND	BSI	No	ICU	Infection rate in the propofol group: 0.2% (3/1,407). Infection rate in the thiopental group: 0.2% (10/5,026). p = NS.

*ICU, intensive care unit; BSI, bloodstream infection; SSI, surgical site infection; SIRS, systemic inflammatory response syndrome; SIR, standardized infection ratio; OR, odds ratio; RR, risk ratio; aOR, adjusted odd ratio; 95% CI, 95% confidence interval; ND, not described in publication; NS, not significant.
†Use of propofol without antimicrobial additives.
‡Use of antimicrobial drug prophylaxis for the procedure.
§Hospital unit where the studies were conducted.
¶Ventilator-associated pneumonia, urosepsis, BSI, catheter-related infections, and others.

Technical Appendix Table 2. Manufacturers' guidelines based on the instructions given by the US Centers for Disease Control and Prevention, US Food and Drug Administration, and the American Association of Anesthesiologists (*11*).

Manufacturers' guidelines	Other recommendations by the CDC and FDA
• Strict aseptic technique must always be used when handling sterile injectable medications<br>• Propofol should be inspected before use for particulate matter, discoloration, or evidence of separation of the emulsion.<br>• Do not use if contaminated.<br>• Fill syringes or spike the vial immediately before administration to each patient.<br>• Disinfect the rubber stopper with 70% isopropyl alcohol.<br>• Discard unused portions within 6 h of filling syringes or 12 h after spiking a large volume vial for infusion.	• Vials of propofol and prefilled syringes are intended for single use (i.e., one patient).<br>• Infusion from prefilled syringes or vials must begin with 6 h of opening/filling the syringe.<br>• Propofol that is infused directly from a large volume (e.g., 100 mL) vial is to be limited to one patient and must be infused within 12 h of opening the vial or spiking the stopper.<br>• Begin infusion immediately after drawing up or opening the medication vial.
